# Parent Opinions About Use of Text Messaging for Immunization Reminders

**DOI:** 10.2196/jmir.1976

**Published:** 2012-06-06

**Authors:** Carolyn Rose Ahlers-Schmidt, Amy K Chesser, Angelia M Paschal, Traci A Hart, Katherine S Williams, Beryl Yaghmai, Sapna Shah-Haque

**Affiliations:** ^1^University of Kansas School of Medicine - WichitaPediatricsWichita, KSUnited States; ^2^University of Kansas School of Medicine - WichitaFamily and Community MedicineWichita, KSUnited States; ^3^Mississippi University for WomenHealth & KinesiologyColumbus, MSUnited States; ^4^Wichita State UniversityPsychologyWichita, KSUnited States; ^5^University of Kansas School of Medicine - WichitaInternal MedicineWichita, KSUnited States

**Keywords:** Provider-patient communication, child immunizations, text message

## Abstract

**Background:**

Adherence to childhood immunization schedules is a function of various factors. Given the increased use of technology as a strategy to increase immunization coverage, it is important to investigate how parents perceive different forms of communication, including traditional means and text-message reminders.

**Objective:**

To examine current forms of communication about immunization information, parents’ satisfaction levels with these communication modes, perceived barriers and benefits to using text messaging, and the ideal content of text messages for immunization reminders.

**Methods:**

Structured interviews were developed and approved by two Institutional Review Boards. A convenience sample of 50 parents was recruited from two local pediatric clinics. The study included a demographics questionnaire, the shortened form of the Test of Functional Health Literacy for Adults (S-TOFHLA), questions regarding benefits and barriers of text communication from immunization providers, and preferred content for immunization reminders. Content analyses were performed on responses to barriers, benefits, and preferred content (all Cohen’s kappas > 0.70).

**Results:**

Respondents were mostly female (45/50, 90%), white non-Hispanic (31/50, 62%), between 20–41 years (mean = 29, SD 5), with one or two children (range 1–9). Nearly all (48/50, 96%) had an S-TOFHLA score in the “adequate” range. All parents (50/50, 100%) engaged in face-to-face contact with their child’s physician at appointments, 74% (37/50) had contact via telephone, and none of the parents (0/50, 0%) used email or text messages. Most parents were satisfied with the face-to-face (48/50, 96%) and telephone (28/50, 75%) communication. Forty-nine of the 50 participants (98%) were interested in receiving immunization reminders by text message, and all parents (50/50, 100%) were willing to receive general appointment reminders by text message. Parents made 200 comments regarding text-message reminders. Benefits accounted for 63.5% of comments (127/200). The remaining 37.5% (73/200) regarded barriers; however, no barriers could be identified by 26% of participants (13/50). Parents made 172 comments regarding preferred content of text-message immunization reminders. The most frequently discussed topics were date due (50/172, 29%), general reminder (26/172, 26%), and child’s name (21/172, 12%).

**Conclusions:**

Most parents were satisfied with traditional communication; however, few had experienced any alternative forms of communication regarding immunizations. Benefits of receiving text messages for immunization reminders far outweighed the barriers identified by parents. Few barriers identified were text specific. Those that were, centered on cost if parents did not have unlimited texting plans.

## Introduction

Adherence to childhood immunization schedules is likely a function of various factors, including parents’ health literacy skills [1-6], immunization knowledge [7,8], and perceived quality of patient–provider communication [9]. Other aspects of immunization communication, including parents’ preferences and perceived barriers, may also influence adherence to childhood vaccination schedules.

Understanding immunization communication from the parents’ perspective should help researchers and practitioners identify specific problems and needs, which might vary among groups. For instance, there is some indication of knowledge gaps among low-income mothers regarding vaccinations, the intended purposes of these immunizations, and appropriate vaccination schedules [7,8]. Determining what these and other parents’ preferences are for receiving vaccination information, as well as identifying perceived communication barriers and needs, might help researchers and practitioners design more effective interventions.

It is possible that certain aspects of communication influence adherence to vaccination schedules. For instance, a recent study [10] investigated the relationship between parents’ health literacy skills and their children’s vaccination status. Early vaccination status did not show a significant association; however, the findings indicated a significant difference in vaccination status at 3 months and 7 months of age. These results highlight the importance of health literacy skills for subsequent vaccinations. Although beyond the scope of the study [10], questions remain about the extent that immunization communication (including appointment reminders) played a role, and whether specific aspects of immunization communication influenced the study results.

Traditionally, medical providers have used face-to-face interaction, pamphlets or handouts, and vaccination appointment reminders (eg, phone calls) as forms of immunization communication [11,12]. The use of technology to provide information, particularly vaccination appointment reminders, has increased over the past few years to include computer-generated auto-dialer phone calls, automated letters and postcards [11], and email reminder programs [12,13]. More recently with the passage of the Meaningful Use rule [14], medical providers with electronic health records (EHRs) are exploring issuing health maintenance reminders. Some success has been found with EHRs in capturing vaccination opportunities in a pediatric population [15] and issuing influenza reminders in older adults [16].

Another use of technology for communicating immunization information is mobile phone text-messaging systems, known as short message services (SMS). While mobile phones are a common commodity across age, gender, and socioeconomic groups [17], SMS-based interventions are in various stages of development and use. Immunization reminders delivered by text-message interventions have shown promising results in some populations [18]. Although some studies have found support for text-message programs from parents of teens [19-22], others have found mixed reactions from medical practitioners [23] as well as organizational barriers and logistical issues that need to be addressed [24].

The impact of text-message reminders on adherence to childhood vaccination schedules continues to be explored. More information is needed to inform the researcher and practitioner of the parents’ perspectives regarding this communication form, preferences they might have, and how text messaging might influence adherence to childhood vaccinations. The current study aims to address this gap.

The purpose of this study is to explore immunization communication utilizing text messages from the parents’ perspective. Because it is critical that children are vaccinated as early as possible in order to avoid vaccine-preventable diseases [10], understanding what might work best from the parents’ viewpoint would be helpful. The findings will contribute to timely and beneficial use of translational immunization and technology research to address adherence to childhood immunization schedules.

## Methods

As part of a feasibility study to determine acceptance among parents of text messages for child immunization reminders, a series of parent interviews were developed and approved by two local Institutional Review Boards. The interview guide was developed by a team with expertise in the areas of pediatric infectious disease, health communication, health disparities, community psychology, and human factors psychology. The guide included informed consent, a 10-item demographic survey, a script of interview questions, and the shortened form of the Test of Functional Health Literacy for Adults (S-TOFHLA) [25].

A convenience sample of parents was recruited from two local group practice pediatric clinics. Parents were informed about the study by the receptionist or nurse at the clinic. If interested, they filled out a form with contact information. The research team conducted a preliminary phone screening of the parent to ensure eligibility criteria were met. Eligibility criteria included: (1) parent or caregiver of a child age < 2 years; (2) adult (≥ 18 years); (3) use a mobile phone for sending and receiving text messages; (4) English speaking; and (5) able to provide informed consent. Parents with valid contact information who met the inclusion criteria (71/95; 75%) were scheduled for a 30–45 minute in-person interview at the medical school. All interviews were scheduled within 1 week of the screening call. Of the 71 appointments made, 30% (21/71) were no-shows. All participants received a US $35 gift card at the conclusion of the interview to cover time and travel costs.

After obtaining informed consent, a self-report demographic survey was administered. The 10 questions were in multiple-choice format, based on the Centers for Disease Control and Prevention’s Behavioral Risk Factor Surveillance System (BRFSS) questionnaire [21]. Interview questions included experience level with technology and current usage patterns, willingness to receive text messages from their child’s medical providers, and perceived barriers. All questions were open-ended, except relationship to technology (used a 6-point scale) and satisfaction with current communication types (used a 4-point scale). For these questions, participants were given a card with their response options.

Specific objectives were to examine the most prevalent forms of communication about immunization information, parents’ satisfaction levels with these communication modes, and perceived barriers and benefits to using a novel form of communication—text messaging. Participants also provided content ideas for what the text messages should read. Following these interview activities, a follow-up study was performed to identify optimal text message content and comprehension of sample text messages. These procedures are described elsewhere [21].

### Health Literacy Assessment

The S-TOFHLA [25] was administered to consenting parents/guardians. Participants completed the 36-item S-TOFHLA, which takes up to 7 minutes to administer and has both normal and large print versions. Scoring results range from 0 to 36; participants are categorized as having adequate health literacy if their S-TOFHLA score is 23–36, marginal health literacy if their score is 17–22, and inadequate health literacy if their score is 0–16. The S-TOFHLA consists of two sections: instructions for preparation for an upper gastrointestinal (GI) series and a Medicaid application [25]. The Gunning Fog readability levels are 4.3 and 10.4, respectively. The passages are set up using a modified Cloze procedure, where approximately every 6th word has been removed.

### Statistical Analysis

All data were entered into Statistical Package for the Social Sciences (SPSS) 17.0. Frequencies and percentages are reported for categorical data; means and standard deviations are reported for continuous data. In addition, content analysis was performed on the open-ended item responses. Participant respondents were counted and quantified for reporting.

The two questions regarding barriers and benefits of text message-based reminders used emergent coding [26]. This method allows the content itself to determine the categories. Two investigators independently reviewed the content and compiled a list of emerging themes. Next, they compared lists and reconciled any differences in order to develop a final list. The investigators confirmed the reliability (Cohen’s kappa > 0.70) of the results. For the question regarding advantages of using text messaging for reminders, 4 categories were identified and 4 categories were also identified for the question regarding barriers.

To assess responses to the question regarding the type of content text-message reminders should include, *a priori *categories determined by an expert panel were used. There were 16 named content categories plus 1 “other” category. Participant comments were assigned to one of the 17 categories by two researchers independently coding the responses. The investigators confirmed the reliability (Cohen’s kappa > 0.70). Discrepancies were corrected by consensus. Frequencies of individual categories were computed.

## Results

Demographically, the majority of respondents were female (45/50, 90%), white non-Hispanic (31/50, 62%), married (20/50, 40%) or members of an unmarried couple (14/50, 28%), with one or two children (range 1–9). Participant age ranged from 20 to 41 years with a mean age of 29 years (SD 5). Most (28/50, 56%) completed 1–3 years of college and 40% (20/50) described their jobs as “employed for wages” with an annual income (from all sources) below US $20,000 (30/50, 60%). Nearly all participants (48/50, 96%) had an S-TOFHLA score in the “adequate” range. One participant’s health literacy level (1/50; 2%) was identified as “marginal” and another (1/50, 2%) had an “inadequate” score.

**Figure 1:**
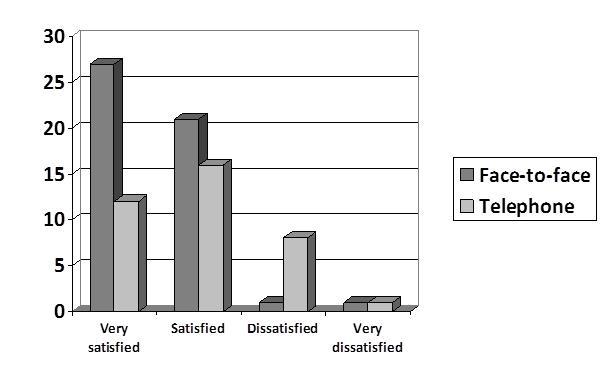
Parent (N = 50) satisfaction with current communication methods with child's health care provider.

Regarding current communication with their child’s physician, all (50/50, 100%) parents engaged in face-to-face contact at the appointments, 74% (37/50) reported communication via telephone, and none of the parents (0/50; 0%) reported using email or text communication. Parental satisfaction with each communication type is illustrated in Figure 1.

Parents reported obtaining the majority of information about immunizations for their child at doctor’s appointments (39/50, 78%); in mailings from the Health Department, Women, Infants and Children program, or Medicaid (12/50, 24%); in mailings from their child’s doctor’s office (11/50, 22%); or the Internet (5/50, 10%). When asked how they know when it’s time to schedule their child’s immunizations, the majority of parents reported being told at their child’s previous appointment and having to remember (38/50, 76%). For older children (> 1 year) some parents relied on memory cues based on their child’s birthday or annual appointment (5/50, 10%) and others relied on the school to let them know (4/50, 8%). One father (1/50, 2%) admitted that his child’s “mommy takes care of that” and if he were on his own he would have no idea what the schedule was.

Almost every participant (49/50, 98%) was interested in receiving immunization reminders by text message and 100% (50/50) were willing to receive general appointment reminders. In addition, 60% (30/50) would be willing to receive lab results by text—although several respondents wanted “only the good results by text, anything bad they should just, like send one that said ‘Lab results are in, please call.’ ” Other suggestions included alerts, such as “We now have the H1N1 vaccine available” (3/50, 6%) and follow-up texts after acute care (2/50, 4%).

For the content analysis, 200 individual comments were coded as relating to either benefits (127/200, 63.4%) or barriers (73/200, 36.5%). Parent comments suggesting benefits of text-message reminders fell into 4 emergent categories: technology, convenience, communication, and general positive (see Table 1). The largest category of comments was “technology” (47/127, 37.0%). Many comments in this category addressed a dislike for talking on the phone or checking voicemail messages.

The barriers to text-message reminders had 4 emergent categories: technology, none identified, communication, and other. “Technology” was also the largest category regarding barriers comments (43/73, 59%). Many of these comments addressed barriers such as if a phone was turned off or lost, while a few comments centered on cost if parents utilized pay-per-text programs. Over ¼ of participants (13/50, 26%) could not identify any barriers. The next most common barrier identified involved communication, with 9 of the 73 comments (12%).

**Table 1:** Content analysis of participant comments (N = 200) about benefits and barriers to text message reminders.

**Category and emergent themes**		**Definition**	**Freq (%)**	**Examples of comments**
**Benefits**				
	Technology	The speed with which information is available, ability to link to other systems/calendars, or other comments related to cellular phone technology.	47 (37.0)	“Text doesn’t waste time and minutes like a phone call,” “You can put it right into appt book in phone,” and “Text will come through later if in bad reception where a phone call won’t.”
	Convenience	Information related to the ease or timeliness of receiving reminders.	35 (27.6)	“Easier,” “quick,” “convenient,” and “I can lock it and go back and check it later.”
	Communication	Includes preferences for, or avoidance of, specific avenues of communication.	23 (18.1)	“Saves time versus phone conversations,” “I respond more to a text,” and “I don’t always check missed calls, but I do check missed texts.”
	General positive	Comments regarding the benefits or the usefulness of reminders in general.	22 (17.3)	“A reminder is good” and “I’m forgetful so I’ll have a written reminder.”
**Barriers**				
	Technology	Comments related to costs, lack of text capabilities, or not being technology-savvy.	43 (58.9)	“You might get charged for text if you don’t have the unlimited plan,” “Turned off phones,” and “Text not working or delayed.”
	None identified	Participant was unable to come up with any negatives to using text messaging.	13 (17.8)	“None,” “none for me,” or “no problems.”
	Communication	Concerns regarding ability to understand text content, its limited characters, the use of abbreviations, or being provided inaccurate information.	9 (12.3)	“Accuracy,” “punctuation,” and “Parents might not understand the text if they don’t know a lot about immunizations.”
	Other	Any item not appropriate for one of the above categories.	8 (11.0)	“Doctor’s office might get overwhelmed with texts coming in” and “forgetting about it.”

Finally, parents were asked to think about the content they would like to see in a text-message immunization reminder (see Table 2). These responses were categorized based on *a priori *categories. The most commonly identified information expected was “date immunizations are due.” The second most frequent response category was “other.” The majority of comments in this category included suggestions of using simple, easy-to-understand language or suggestions of what “not” to include. The only item that fell into the other category that was informational content was doctor’s office location, which was identified by 10% (5/50) of parents.

**Table 2:** Frequency of participant comments (N = 172) about preferred text message immunization reminder information.

**Category (** *a priori* **)**		**Example**	**Freq (%)**
	Date immunizations are due	“When appointment is,” “Appointment tomorrow at 9:15,” “When shots are due.”	50 (29.1)
	Other information	“Location” and “Use numbers—9 months vs nine months.”	31 (18.0)
	General reminder	“Immunization due soon” and “Time to schedule an appointment for shots.”	26 (15.1)
	Child’s name	“Which child” and “Name of child.”	21 (12.2)
	Name of vaccine	“What type of shot” and “Names of vaccines.”	16 (9.3)
	Clinic number	“Phone number for the Doctor” and “If questions contact at this number.”	11 (6.4)
	Child’s age	“Time frame—like 6 month immunizations” and “Baby’s age.”	9 (5.2)
	Doctor’s name	“Doctor’s name” and “Dr. [name].”	3 (1.7)
	Number of shots	“How many shots.”	2 (1.2)
	Name of vaccine-preventable disease	“What the shot prevents.”	1 (0.6)
	Side effects	“Side effects so you can be prepared for after the appointment.”	1 (0.6)
	What the disease can do to my child	“Risks of the disease.”	1 (0.6)
	Full immunization schedule		0 (0)
	How the disease spreads		0 (0)
	Total number of specific vaccine needed		0 (0)
	Who is at risk?		0 (0)
	Where your child is in the specific vaccine series		0 (0)

## Discussion

The purpose of this study was to explore immunization communication among parents to better understand how adherence to childhood vaccinations can be addressed. Specifically, we wanted to identify the most prevalent forms of immunization communication, examine parents’ satisfaction with these communication modes, and—because of the increased use of mobile phone text immunization reminders—explore perceived barriers and benefits to using text messaging.

Parents in our study were most likely to receive communication about immunization information face-to-face at the pediatricians’ office. Most parents were satisfied with this form of communication; however, few had experienced any alternative forms of communication regarding immunizations. Parents indicated less satisfaction with telephone than face-to-face communication. Although several parents reported receiving mailers from their child’s physician, no parents reported email or text message communication. This is consistent with results from a recent survey of physicians conducted in the same metropolitan area. In that study, Chesser et al [27] found that none of the responding pediatricians or family physicians were using email or text messaging to convey immunization reminders to parents; in addition, these physicians were divided in their opinions of the appropriateness of sending text messages to parents [23].

Parents, on the other hand, were interested in receiving text message communication from their child’s physician. This may indicate that text messaging is considered a form of constructive communication from physicians, as suggested by Raine et al [28]. In fact, all participants were willing to receive text-message reminders for general appointments. One mother did not wish to receive immunization-specific text reminders; she reported her child was on an “alternative” immunization schedule, but would not share any more detail on what that meant.

Parents were able to identify nearly twice the benefits to receiving text messages as barriers, and most of the barriers identified were not text specific. For example, many comments addressed barriers such as if a phone was turned off or lost which would also apply to phone calls or voicemails to mobile phones. Most of the comments that could be applied exclusively to text messaging seemed centered on cost if parents did not have unlimited texting plans. However, these comments were meant generally and did not necessarily apply to the participant making them. Several parents who had unlimited text messaging capabilities stated, “It might get expensive for people who have to pay for each text.” This barrier is not insurmountable as a recent survey of low-income parents found 81% of the 167 parents with text messaging had an unlimited text plan [19].

The second most common barrier identified was communication. This is not surprising as discrepancies between physicians and patients in general literacy levels can result in misunderstanding of written handouts and have resulted in efforts to rewrite materials to improve their usefulness to patients [29]. Whereas health literacy is one explanation for poor patient understanding of medical information, determining who has low health literacy presents a challenge to physicians. Physicians can enhance understanding by assessing health literacy at new patient visits, refraining from use of medical terminology, drawing a picture, limiting information, assessing understanding using a teach-back method, and being respectful and sensitive in order to empower patients [30,31].

In the current study, we first asked parents to free-associate the content they would like to see in a text-message reminder and later, in the follow-up study, we asked them to organize cards with sample content into critical, clarifying, or unnecessary information to include in a text message [21]. The top 6 critical content items selected from the card sort were in the same categories mentioned by interviewed parents as content they would like to see in a text-message immunization reminder. This suggests some reliability in the responses given by parents. In addition, these results were consistent with other published studies [20,22]. As with these previous studies, participants in the current study suggested that the information be short, simple, and personalized. One item we had not anticipated was the importance of the clinic address to parents. This information may be most important to include in reminders for the initial immunization appointment or for certain populations and should be further investigated.

It is important to remember that the limitation of text messages to 160 characters also limits the information that can be conveyed with this technology. While text-message immunization reminders may have the capability to enhance parental understanding of immunization schedules, this is probably not the most appropriate mode to address parents’ attitudes, knowledge, and trust regarding vaccines. Wu et al identified knowledge gaps in mothers’ understanding of vaccinations and suggested mothers would benefit from discussions regarding risks and benefits of vaccines during prenatal care [32].

### Limitations

While this study was important for understanding immunization communication from parents’ viewpoints, there are limitations that should be considered. First, self-reported preferences, behaviors, and experiences might be an issue as some parents may have inaccurately responded to interview questions. Socially desirable responses might have impacted the results.

Generalizability of the study findings might also be an issue due to the small sample size. Participating parents came from a single Midwestern urban city, had relatively high education and health literacy levels, spoke only English, and were proficient with text messaging. Thus, participants in this study might not have been representative of most parents, even in the targeted city. Further research is needed to examine immunization communication in larger samples and among diverse groups of parents. Results might differ among those with less text messaging experience, marginal or low health literacy, and varied ethnicity [20] and language.

### Conclusions

The goal of this study was to provide information that will lead to the development of parental interventions and programs to help improve adherence to childhood immunization schedules. The research findings are being used to design an intervention versus control model to be used in a future study assessing impact of text messages on vaccine schedule compliance. Based on the premise that children’s health is influenced by various parental factors, including parents’ health literacy skills, knowledge, and perceptions of patient–provider communication about immunization information, these results may be used to help providers and researchers effectively promote early adherence, services, and immunization programs directly to parents.

### Practice Implications

While other studies have assessed whether various types of immunization reminders improve immunization rates [33] more information is needed. Limited information existed regarding what communication methods were preferred and what issues might exist according to parents. With the increased use of text messaging as a form of communication, it was important to know whether this was a viable form of immunization communication with parents. The current study addressed these needs and provided greater understanding of immunization communication from the parents’ perspective. The findings provide a basis for further research and text-message interventions that could address adherence to childhood immunization schedules.

The findings of this study and others [19-22] suggest an overwhelming support for text-message immunization reminders from parents who utilize text messaging. Text messaging may not only be a viable tool for increasing immunization compliance in children, but may actually be the preferred tool of some subgroups of parents. In general, physicians should consider utilizing advances in SMS-based technology to enhance communication regarding immunizations—a benefit seen in other health promotion programs including diabetes management [34] and weight loss [35].

## Abbreviations

BRFSS: Behavioral Risk Factor Surveillance System
GI: gastrointestinal
SMS: short message service
SPSS: Statistical Package for the Social Sciences
S-TOFHLA: Test of Functional Health Literacy for Adults (shortened form)
